# Incidence of aplastic anemia and agranulocytosis in Latin America — The LATIN study

**DOI:** 10.1590/S1516-31802005000300002

**Published:** 2005-05-02

**Authors:** Eliane Maluf, Ricardo Pasquini, José Eluf, Frederico Rafael Moreira, Alexandre Biasi Cavalcanti, Íria Ruriko Okano, Roberto Passeto Falcão, Marimília Teixeira Pita, Sandra Regina Loggetto, Luiz Gastão Rosenfeld, Irene Gyongyvér Heidemarie Lorand-Metze

**Keywords:** Aplastic anemia, Agranulocytosis, Incidence, Risk factors, Granulocytes, Anemia aplástica, Agranulocitose, Incidência, Fatores de risco, Granulócitos

## Abstract

**CONTEXT AND OBJECTIVE:**

Aplastic anemia and agranulocytosis are rare but life-threatening disorders, often caused by drugs and other environmental exposures. Reported incidence of these diseases seems to vary between different geographic regions, and few data on their incidence are available for Latin American countries. The aim of this work is to determine the incidence of agranulocytosis and aplastic anemia in Brazil.

**DESIGN AND SETTING:**

Incidence study. Seven centers took part in the pilot phase, so as to represent all Brazilian regions.

**METHODS:**

Each center conducted an active search for new cases in a defined region by means of regular contacts with all hematologists, main clinical laboratories and clinicians in hospitals of the region.

**RESULTS:**

74 patients with aplastic anemia and 16 with agranulocytosis were identified. Patients with agranulocytosis had a median age of 31 years (interquartile range, IQR: 12.5-48.2); 32.2% were male and 81.2% were white. The median age of aplastic anemia patients was 21 years (IQR 15.0-35.2); 62.2% were male, 50.0% were white and 39.2% mulatto. The incidence of agranulocytosis was estimated to be 0.5 cases per million individuals per year, ranging from 0.0 to 1.1 cases per million per year between regions. The incidence of aplastic anemia was 2.7 cases per million per year, ranging from 1.1 to 7.1 cases per million per year between regions.

**CONCLUSIONS:**

Aplastic anemia and agranulocytosis are rare diseases in Brazil. However, there is considerable variability in their incidences between different regions.

## RESUMO

**CONTEXTO E OBJETIVO:**

A anemia aplástica e a agranulocitose são doenças raras, entretanto freqüentemente letais. Muitas vezes são causadas por medicações e outras exposições ambientais. A incidência dessas doenças parece variar consideravelmente entre diferentes regiões geográficas, e poucos dados sobre a incidência são disponíveis para os países da América Latina. O objetivo deste trabalho é determinar a incidência de anemia aplástica e agranulocitose no Brasil.

**TIPO DE ESTUDO E LOCAL:**

Estudo de incidência. Sete centros participaram da fase piloto do estudo representando as cinco regiões brasileiras.

**MÉTODOS:**

Cada centro realizou busca ativa por novos casos em uma região definida, por meio de contatos regulares com todos os hematologistas, principais laboratórios clínicos e clínicos de hospitais de sua região.

**RESULTADOS:**

Foram identificados 74 casos de anemia aplástica e 16 casos de agranulocitose. A mediana de idade dos pacientes com agranulocitose foi de 31 anos (intervalo inter-quartil - IIQ 12,5 - 48,2), 32,2% eram do sexo masculino e 81,2% eram da raça branca. A mediana de idade dos pacientes com anemia aplástica foi de 21 anos (IIQ 15,0-35,2), 62,2% eram do sexo masculino, 50,0% da raça branca e 39,2% da raça parda. A incidência de agranulocitose foi estimada em 0,5 casos/milhão de habitantes/ano, variando de 0,0 a 1,1 caso/milhão de habitantes/ano entre as diferentes regiões brasileiras. A incidência de anemia aplástica foi de 2,7 casos/milhão de habitantes/ano, variando de 1,1 a 7,1 casos/milhão de habitantes/ano entre as diferentes regiões.

**CONCLUSÕES:**

A anemia aplástica e a agranulocitose são doenças raras no Brasil. Entretanto existe considerável variabilidade na incidência destas doenças entre as diferentes regiões brasileiras.

## INTRODUCTION

Agranulocytosis is defined as an absolute neutrophil count of less than 500/μl. It is a rare condition, with an estimated incidence of 1 to 10 cases per million individuals per year,^1-5^ and it is associated with a case-fatality rate of 6-10%.^3,6^ In 70% of the cases, an association with the use of medications is established.^3^

Aplastic anemia is also a low-incidence disease, ranging from 0.5 to 4 cases per million individuals per year.^3,5,7^ However, its clinical evolution is more severe, involving higher case-fatality and requiring more complex therapeutic interventions.^8-12^

The LATIN study has the goals of estimating the incidence of aplastic anemia and agranulocytosis in Latin American countries and identifying the risk factors for these diseases. The study was planned to have two phases: an initial phase ("pilot phase") and the main phase. The pilot study began in April 2002 and was completed in April 2003, while the main phase is planned to end in August 2006. The pilot phase of the study was aimed at assessing the methodology proposed for conducting the study and identifying potential problems and difficulties, thereby allowing the identification of solutions for conducting the main phase of the study.

In the present paper, we report on the incidence of agranulocytosis and aplastic anemia in Brazil that was observed in the pilot phase of the LATIN Study.

## METHODS

Seven centers took part in the pilot phase: Hospital das Clínicas da Universidade Federal do Paraná (Curitiba, State of Paraná), Instituto de Hemoterapia de Goiânia (Goiânia, State of Goiás), Hemocentro Regional de Juiz de Fora (Juiz de Fora, State of Minas Gerais), Fundação HEMOAM (Manaus, State of Amazonas), HEMOPE (Recife, State of Pernambuco), Faculdade de Medicina da Universidade Federal de São Paulo/Ribeirão Preto (Ribeirão Preto, State of São Paulo), and Hemocentro Regional de Uberaba (Uberaba, State of Minas Gerais). The centers were selected so as to represent all Brazilian regions. In addition to the seven Brazilian centers mentioned above, other centers in Argentina, Colombia and Mexico will take part in the main phase of the study.

In order to estimate the incidence of agranulocytosis and aplastic anemia, each center conducted an active search for new cases throughout its State, with the exception of Ribeirão Preto (State of São Paulo), where the active search region was restricted to adjacent cities. For the purpose of carrying out the active search, each center relied upon one or more study coordinators, who drew up contact lists including all the active hematologists within the region covered, the main clinical laboratories and other clinicians in hospitals in the region. Initially, a document was sent to these professionals that included a summary of the study and a request for their collaboration through reporting possible new cases to the study coordinators. The latter kept up weekly contact with the professionals in the various institutions. Whenever new suspected cases were identified, the study coordinators would head for the centers and conduct the interviews. Only the patients who had been living in the region covered by the research center for more than three months were considered as incident cases (for incidence calculation).

Patients were defined as having agranulocytosis if they were symptomatic, i.e. if they presented a clinical picture consistent with agranulocytosis (e.g. pharyngitis), with granulocytes < 500/mm^3^, compatible bone marrow (excluding other diagnoses), hemoglobin > 10.0 g/dl and platelets > 100,000/mm^3^. Exceptionally, the inclusion of cases in which the myelogram or bone marrow biopsy had not been performed was permitted. In this latter case, laboratory findings of granulocytes < 500/mm^3^ for two consecutive days, with leukocytes < 3,000/mm^3^ in the consecutive analysis (increased to > 1,000/mm^3^ within 30 days), were required.

The cases of aplastic anemia were defined according to the presence of two or more of the following criteria: 1) leukocytes < 3,500/mm^3^; 2) platelets < 50,000/mm^3^; 3) hemoglobin < 10.0 g/dl or hematocrit < 30%. Additionally, the bone marrow evaluation had to be consistent with the diagnosis, demonstrating hypocellularity, but excluding fibrosis, lymphomatous or carcinomatous leukemic infiltration, or hypocellular myelodysplasia. Although bone marrow evaluation by biopsy was recommended, a myelogram was accepted as an alternative.

All the cases of agranulocytosis and aplastic anemia surveyed were submitted to analysis by an Independent Event Validation Committee (IEVC), composed of hematologists with broad experience in the field. The IEVC had access to laboratory results, bone marrow tests (biopsy or myelogram) and summaries of the patients’ clinical histories. However, information concerning exposure was not provided to the IEVC. Only the validated cases were included in the study.

Data were collected by the study coordinators, who were nurses or pharmacists. All study coordinators underwent a standard training program in the study coordination office.

### Data Management

Data entry was performed through electronic capture via the Internet, which consisted of a form using HTML resources that was similar in appearance to the manual filing form, but had the advantage of giving the possibility of selecting data coded in available lists. A few fields were restrictive, such that values outside of pre-established limits could not be entered, and some fields were interlinked, such that divergent information was not allowed.

### Data Analysis

The quantitative variables were described through means and standard deviations or medians and quartiles; the categorial variables were expressed as absolute and relative frequencies.

To determine the incidence, the total population of the regions where active search was carried out was used as the denominator. The population count was obtained through the 2000 census by the *Instituto Brasileiro de Geografia e Estatística* (IBGE) [Brazilian Institute of Geography and Statistics].^13^

## RESULTS

During the pilot study, 74 cases of aplastic anemia and 16 cases of agranulocytosis were included.

The patients with agranulocytosis had a median age of 31.0 years, though great variability was found, as demonstrated by an interquartile range of 12.5 to 48.2. Most of the patients were female (68.8%) and white (81.2%) (Table 1).

**Table 1:** Demographic characteristics of the patients with agranulocytosis and aplastic anemia in the pilot phase of LATIN study*

	Agranulocytosis	Aplastic anemia
Gender n (%)		
Male	5 (31.2)	46 (62.2)
Female	11 (68.8)	28 (37.8)
Race n (%)		
White	13 (81.2)	37 (50.0)
Black	—	6(8.1)
Mulatto	3 (18.8)	29 (39.2)
Indigenous	—	2 (2.7)
Age in years - median (IQR^a^)	31 (12.5-48.2)	21 (15.0-35.2)

a *IQR = interquartile range*

* *data obtained from April 2002 to April 2003.*

The median age of the patients with aplastic anemia was 21.0 years, with an interquartile range of 15.0 to 35.2 years. Most patients (62%) were male. Among the patients with this disease, 50.0% were white, 39.2% were mulatto, 8.2% black and 2.7% indigenous (Table 1).

A total incidence of 0.5 cases of agranulocytosis per million individuals per year and 2.7 cases of aplastic anemia per million individuals per year was observed. There was considerable variation in the incidence values observed between centers (Table 2).

**Table 2:** Incidence of agranulocytosis and aplastic anemia in six different Brazilian centers registered in the pilot phase of LATIN study*

State	Agranulocytosis		Aplastic anemia	
	Cases	Incidence (cases/million/year)	Cases	Incidence (cases/million/year)
Paraná	7	0.7	25	2.6
Ribeirão Preto (region)	3	1.1	2	1.1
Pernambuco	1	0.1	23	7.1
Minas Gerais	4	0.3	18	2.4
Goiás	0	0.0	4	1.4
Amazonas	1	0.5	2	1.7
**Total**	**16**	**0.5**	**74**	**2.7**

* *Data obtained from April 2002 to April 2003 and incidences calculated on a population basis as indicated by 2000 Brazilian Census.^13^*

## DISCUSSION

The analysis of the data generated during the pilot phase of the LATIN study enabled the obtaining of preliminary values for the incidence of aplastic anemia and agranulocytosis, derived from seven centers in five Brazilian regions. We observed marked variability in incidence between the regions, and this pattern resembled the variability in incidence between countries that has been reported from previous studies.^14,15^

The incidence of aplastic anemia observed in the present study, 2.7 cases/million individuals/year, is similar to what was previously reported from a study in southern Brazil (2.4 cases/million individuals/year),^7^ and in the International Agranulocytosis and Aplastic Anemia Study (IAAAS) (2.0 cases/million individuals/year).^14^ The incidence of aplastic anemia reported from studies in Mexico and Thailand was higher: 3.9 and 4.1 cases/million individuals/year, respectively.^15,16^

Aplastic anemia was more frequent among males, as observed in the Thai study.^15^ Prior studies found two incidence peaks of aplastic anemia: within the age group of 15 to 24 years and above the age of 60 years.^7,15^ In the pilot phase of the present study, this pattern was not found but rather, a more homogeneous distribution among age groups. Nevertheless, these initial results should be interpreted cautiously, since they come from a small number of patients.

The incidence of agranulocytosis was 0.5 cases/million inhabitants/year in this study. In agreement with previous reports, the cases were most often females. On the other hand, an unexpectedly high frequency was observed among younger individuals: the median age was 31 years, whereas it was over 50 years in the IAAAS.

We based our definition of agranulocytosis and aplastic anemia on previous studies,^3,15^ in order to make comparisons. Allowing the inclusion of aplastic anemia cases diagnosed on the basis of myelograms probably increased the sensitivity of the study because it is not current practice to perform biopsies in all cases all over the country. The incidence of agranulocytosis reported previously was higher than in the present study, ranging from 0.8 cases/million individuals/year, in the Thai study,^15^ to 3.4 cases/million inhabitants/year, in IAAAS.^14^ However, retrospective data obtained in a Brazilian study has also suggested a lower incidence of 0.44 to 0.82 cases/million inhabitants/year.^1^ We believe that the low incidence of agranulocytosis reported in the pilot phase of this study may be due, at least in part, to physicians’ failure to diagnose the disease in symptomatic patients, for instance those with sore throat secondary to agranulocytosis. This problem is more likely to occur in regions where access to good medical care is inadequate. However, we cannot rule out the possibility that some cases with an established agranulocytosis diagnosis were not identified by the investigators. This is a possible flaw in the study and, on this basis, we decided to implement measures to increase the sensitivity of case identification. One example of these efforts was a request made to the National Agency for Sanitary Surveillance (Anvisa) for it to encourage physicians to report any suspected cases to the study coordinators.

Following the pilot phase, we have concluded that a larger study is feasible and we have made two major changes to the protocol. Firstly, we have restricted the area covered by each center’s active search, so as to include only the cities with better medical systems. Secondly, we have revised the case report forms, which now are simpler and more objective. Finally, as planned initially, other centers in Argentina, Colombia and Mexico have joined the study.

Agranulocytosis and aplastic anemia are rare in Brazil. However, the incidence of both diseases varies considerably between different regions. A large observational study, such as the ongoing main phase of the Latin Study, is needed to better establish the incidence of these conditions in Latin American countries.

### Appendix: Investigators in The Latin Study

#### Steering Committee:

Nelson Hamerschlak (co-principal investigator) — Centro de Pesquisa Clínica, Instituto Israelita de Ensino de Pesquisa Albert Einstein, São Paulo, Brazil; Ricardo Pasquini (co-principal investigator), Eliane Maluf (co-principal investigator) — Hospital das Clínicas, Universidade Federal do Paraná, Curitiba, Brazil; José Eluf-Neto, Departamento de Medicina Preventiva, Faculdade de Medicina, Universidade de São Paulo, São Paulo, Brazil.

#### Executive Committee:

Iria Ruriko Okano, Alexandre Biasi Cavalcanti — Centro de Pesquisa Clínica, Instituto Israelita de Ensino de PesquisaAlbert Einstein, São Paulo, Brazil.

#### Independent Committee for Event Validation:

Marimília Teixeira Pita (Centro de Hematologia de São Paulo), Roberto Passeto Falcão (Faculdade de Medicina da USP-Ribeirão Preto), Luiz Gastão Rosenfeld Centro de Hematologia de São Paulo), Sandra Regina Loggetto (Centro de Hematologia de Sao Paulo), Irene Gyongyvér Heidemarie Lorand-Metze (Universidade de Campinas-UNICAMP).

#### Data Management:

Ruy Guilherme Rodrigues Cal, Marcos Rodrigues Gouvea, Centro de Pesquisa Clinica-Instituto Israelita Albert Einstein de Ensino e Pesquisa.

#### Statistician:

Frederico Rafael Moreira, Centro de Pesquisa Clinica-Instituto Israelita Albert Einstein de Ensino e Pesquisa.

#### Advisory Board:

David W. Kaufman (Slone Epidemiology Center, Boston University, Boston, MA, USA.), Samuel Shapiro (Slone Epidemiology Center, Boston University, Boston, MA, USA.), Joan-Ramon Laporte (Fundacio Institut Catala de Farmacologia, World Health Organization Collaborating Centre for Research and Training in Pharmacoepidemiology, Barcelona, Spain), Ricardo Sesso (Centro de Pesquisa Clinica-Instituto Israelita Albert Einstein de Ensino e Pesquisa), Celso Carlos de Campos Guerra (Centro de Hematologia de São Paulo).

#### Centers and Principal Investigators:

Hospital das Clínicas, Universidade Federal do Paraná, Curitiba, Brazil: Eliane Maluf, Ricardo Pasquini; Instituto de Hemoterapia de Goiânia, Goiânia, Brazil: César Leite Sant’Anna, César Bariani, Geraldo Sant’Anna Cunha Jr.; Hemocentro Regional de Juiz de Fora, Juiz de Fora, Brazil: Daniela de Oliveira Werneck Rodrigues, Abrahão Hallak Neto; Fundação HEMOAM, Manaus, Brazil: Leny Nascimento da Motta Passos, Ruth Perdiz; Fundação HEMOPE, Recife, Brazil: Erika Oliveira de Miranda Coelho, Raul Melo; Faculdade de Medicina da Universidade de São Paulo/Ribeirão Preto, Ribeirão Preto, Brazil: Rodrigo do Tocantins Calado, Maria Carolina Tostes Pintão; Hemocentro Regional de Uberaba, Uberaba, Brazil: Hélio Moraes de Souza, Ana Marcela Rojas Fonseca; Hospital de Clínicas José de San Martín, Buenos Aires, Argentina: Daniel Goldenberg; Hospital San José Tec. de Monterrey, Monterrey, Mexico: José Rafael Borbolla, Enrique Baez de la Fuente; Hospital Central Policia Nacional, Bogotá, Colombia: Magali de Los Rios de Acevedo, Alexander Monsalve.

### Role Of The Funding Source

Sanofi-Aventis and Boehringer-Ingelheim had no role in study design, data collection, data analysis, data interpretation, or writing of the report.
